# Modified Distance Transformation for Image Enhancement in NIR Imaging of Finger Vein System

**DOI:** 10.3390/s20061644

**Published:** 2020-03-16

**Authors:** Krzysztof Bernacki, Tomasz Moroń, Adam Popowicz

**Affiliations:** 1 Department of Electronics, Electrical Engineering and Microelectronics, Silesian University of Technology, Akademicka 16, 44-100 Gliwice, Poland; kbernacki@polsl.pl; 2 Department of Cybernetics, Nanotechnology and Data Processing, Silesian University of Technology, Akademicka 16, 44-100 Gliwice, Poland; tomasz.moron@polsl.pl

**Keywords:** image processing, person identification, infrared imaging, image sensors

## Abstract

Most of the current image processing methods used in the near-infrared imaging of finger vascular system concentrate on the extraction of internal structures (veins). In this paper, we propose a novel approach which allows to enhance both internal and external features of a finger. The method is based on the Distance Transformation and allows for selective extraction of physiological structures from an observed finger. We evaluate the impact of its parameters on the effectiveness of the already established processing pipeline used for biometric identification. The new method was compared with five state-of-the-art approaches to features extraction (position-gray-profile-curve—PGPGC, maximum curvature points in image profiles—MC, Niblack image adaptive thresholding—NAT, repeated dark line tracking—RDLT, and wide line detector—WD) on the *GustoDB* database of images obtained in a wide range of NIR wavelengths (730–950 nm). The results indicate a clear superiority of the proposed approach over the remaining alternatives. The method managed to reach over 90% identification accuracy for all analyzed datasets.

## 1. Introduction

One of the methods of person verification utilizes the near-infrared (NIR) images of the finger vascular system, which is proved to contain a set of features unique for each human. External features, like a fingerprint, finger shape, skin folds and lunulas are accompanied by the internal features—the structure of a vascular system [1]. Finger tissues and blood have different absorption coefficients for various light wavelengths which phenomenon allows for observation of both types of features in NIR images. While the visible cannot penetrate inside the body showing only the skin, the NIR reveals and distinguishes internal structures from the external ones. This is because the light in a range of 700–1000 nm is strongly absorbed by oxidized haemoglobin in veins [2] and lowly by the tissues [3]. This method of identification has been already described well in rich literature [4].

Currently, most of the methods dedicated for image processing in such imaging, focus on good isolation of the vascular system from an image to be further processed by suitable classification techniques [5,6,7]. However, we are convinced that the external features of a finger can further improve the verification reliability. Unfortunately, none of the methods of initial processing of raw images concentrates on proper extraction of both types of features. This is due to the fact that internal and external parts of a finger are somewhat different, showing varying structural characteristics (sizes, shapes, range of intensities in the image, etc.) which would require a flexible processing schema. The method presented in this paper is the first such approach that tries to include and enhance the most representative features in the image to improve the accuracy of person identification.

## 2. Materials and Methods

### 2.1. Distance Transformation

The original idea of Distance Transformation (*DT*) was presented in [8]. The main goal of the method is to find the distance from a given pixel to all other. The main advantage of this approach was the very short time of execution, much shorter than other methods based on computing the Euclidian distance. Several modifications of the method have been already presented. One of them included calculation of topological distance, i.e., counting intensity differences instead of spatial difference between pixels positions for colorization purpose [9]. A combination of the topological and spatial distances can be found in [10]. This approach was adopted by the authors in the estimation of image quality of the finger vein vascular system [11].

**Figure 1:**
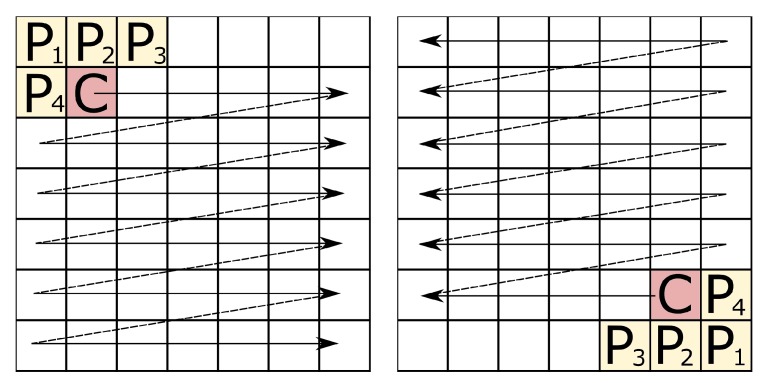
The L-shaped mask used in the original Modified Transformation and its sliding manner the double-scan technique.

In its simplest form, the Distance Transformation of an image is calculated as follows. Initially the distance matrix is initialized with infinities in all pixels excluding the object’s pixels—for them the distance equals 0. Next, the distance matrix is swept twice (this is so-called double-scan algorithm) with a special L-shaped mask as presented in Figure 1. In each step of the mask, one replaces the distance of a central pixel ($d_{C}$) pixel with its new values using the formula:

(1) $$d_{C}=min\left\{d_{P_{1}}+\sqrt{2},d_{P_{2}}+1,d_{P_{3}}+\sqrt{2},d_{P_{4}}+1,d_{C}\right\}$$

After two passes, the distances in each pixel are rough estimations of the Euclidean distance to the closest object’s pixel.

We propose the Modified Distance Transformation (MDT) in which we replace the formula above so that it operates on the intensities of pixels rather than on binary image:

(2) $$d_{C}=min_{\left(i=1,2,3\right)}\{d_{P_{i}}+\alpha |I_{C}-I_{P_{i}}\left|\right\},$$

where $I_{C}$ and $I_{P_{i}}$ stands for the intensity of a pixel at central position (*C*) and at $P_{i}$ in the mask. The α is the trigger which allows for distance growth only if the intensity of a central pixel is higher then at neighbouring pixel (i.e., α=1 if $I_{A}-I_{P_{i}}>0$). As a result, the distance will grow along intensity gradients only for positive slopes. This triggered approach was adopted to better fit to the characteristics of veins in images, since their neighbourhood is always brighter.

In our application of MDT we select a pixel in image with its local N×M neighbourhood (Ω). In this small region we perform MDT so that each pixel in Ω receives its modified distance from the central pixel. The new value of a pixel in MDT-enhanced image receives the mean of distances calculated within Ω window. If the intensity gradients in the window are small, no visible structures are detected and so the result of *MDT* is low. On the other hand, if a pixel is within some well distinguishable structure, the result of *MDT* is high. It is even higher when the central pixel is visibly dimmer than its neighbourhood. By changing window size (in both *x* and *y* directions) we can obtain differing filtering outcomes that can expose various finger’s structures: internal, external and mixed.

**Figure 2:**
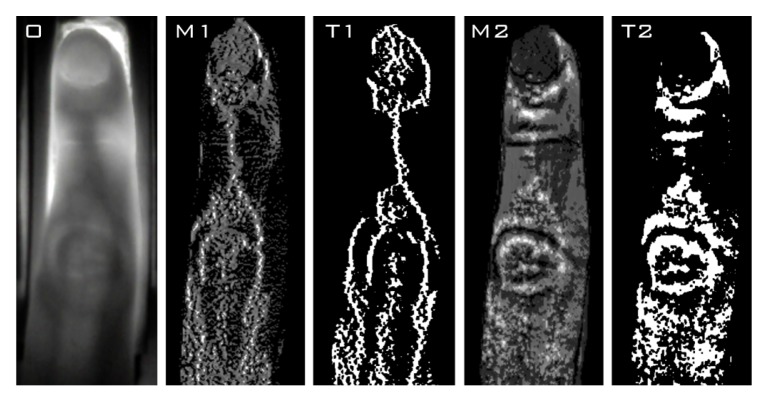
Exemplary results of processing images with *MDT* (before and after binarization) using two sizes of operational window (9×3 pixels on the left and 9×19 pixels on the right side). *O*—original image, *M1/M2*—image processed with MDT, T1/T2—thresholded image (after Otsu binarization).

**Figure 3:**
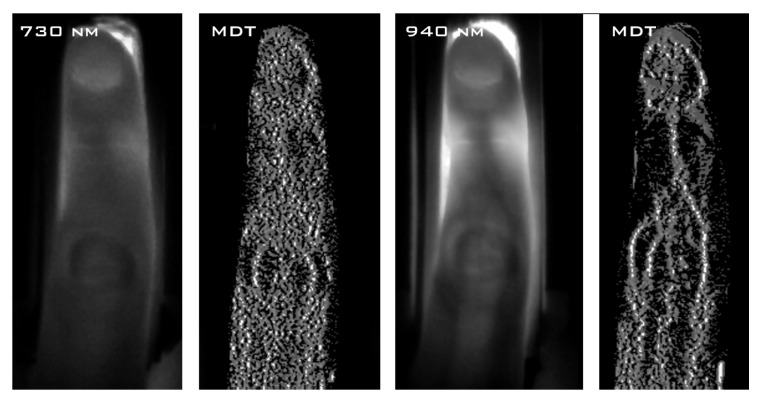
Comparison of *MDT* processing of images registered at 730 and 940 nm. From the left: original image 1 (730 nm), processed image 1, original image 2 (940 nm) and processed image 2. In both cases the operational window of 9×3 pixels was utilized.

We present examples of images obtained from consecutive steps of image processing using *MDT* in Figure 2 for two sizes of operational window. The original image (*O*) and the results of *MDT*-enhancement (*M1/M2*) are presented together with respective Otsu’s binarization [12] outcomes (*T1/T2*). Clearly, it can be seen that depending on the utilized window significantly different internal and external structures are enhanced and subsequently segmented. In Figure 3 we also show the results of *MDT* processing of data obtained at 730 nm and 940 nm for the same size of the window (9×3 pixels). In the longer wavelengths the vein system is much better visible both before and after processing with *MDT*.

In our previous research [11] the *MDT* was used to estimate the image sharpness and to assess if an image is in-focus, currently we use it for image enhancement applied before segmentation phase. We extend the range of sizes of a running, operational window in which the distance transformation operations are performed (previously a fixed window size was used). The mentioned quality measure of a pixel is now assumed as a filtered intensity. Therefore, the proposed filtering schema gives each pixel a value which depends on the relations between intensities of neighboring pixels in the operational window.

### 2.2. Biometric System

The evaluation of method’s effectiveness was conducted using the biometric database *GustoDB* [11,13,14] which contained a total number of over 34,000 images in nine wavelengths between 730 nm and 950 nm, obtained from 107 volunteers, for both left and right hands. Our apparatus consisted of (1) ten replaceable finger pads with NIR diodes (wavelengths mentioned above), (2) CCD camera with CCTV lens allowing for imaging with 3.8 pix/mm scale and (3) an hand-box isolated from external light and with black walls (to reduce NIR light scattering) in which the images are acquires. Although we concentrate only on finger images, the system was prepared to allow for taking pictures of whole hand. Additionally, the Arduino micro controller was used to control the intensities of NIR diodes so that the images in the camera are not saturated. Several most important parameters of the equipment used during construction of the database are given in Table 1. For more details of *GustoDB* see the original work [13].

**Figure 4:**
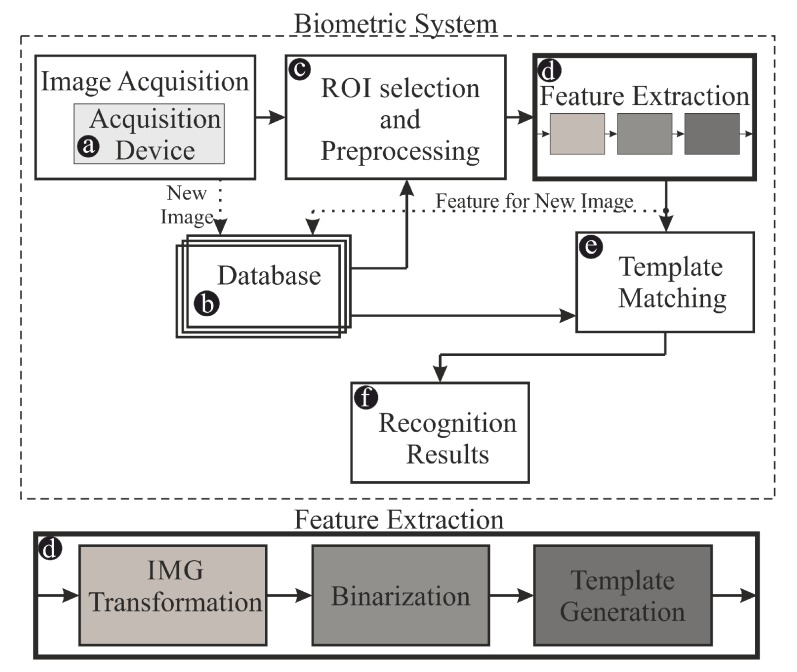
The overview of the biometric pipeline used in the experiments. The highlighted block—*IMG Transformation*—is the place where the new algorithm is proposed.

The full pipeline used for the identification is presented in Figure 4. Its details are given in our recent work [11]. This biometric system consists of the following parts:

1. image acquisition in a selected wavelength using the CCD webcam with removed infrared filter from the sensor and the illumination utilizing LED NIR diodes,
2. storage of raw data in *GustoDB* database,
3. pre-processing procedures,
4. extraction of the region of interest (ROIs),
5. extraction the features from the ROIs,
6. template matching by calculating the similarity of features extracted from images and the data already collected in the database,
7. making the final decision about recognized personality.

The dotted arrows show a possible path of data flow when building the database (collecting images). During the identification process, this path is not used, expect for the situation when one wants to add new data (i.e., include a new person in the set).

Block (c) of the pipeline (see Figure 4) was implemented using the methods described and commonly used in the literature. Initially, the images are corrected for the offsets resulting from either generation of the dark current in CCD pixels or from the constant bias charge. This is done by taking dark frames (images acquired with the same exposure time and the matrix gain set as for the light frames), averaging them to improve their signal-to-noise ratio, and subtracting averaged dark frame from each taken image. In this procedure, the pixels exhibiting saturation are also identified and interpolated (if they are faulty) or excluded from further analysis if the saturation originated in too high light level. Next, the min-max histogram stretching is done to fit the range of intensities to 8-bit range (0–255). In the final step of pre-processing, the finger is extracted from the image using gradient-based approach presented by authors in [15].

In feature extraction part (d), three sub-blocks are present: image transformation, binarization and template generation. The transformation may utilize several alternative approaches: position-gray-profile-curve (*PGPC*) [16], maximum curvature points in image profiles (*MC*) [17], Niblack image adaptive thresholding (*NAT*) [18], repeated dark line tracking (*RDLT*) [5], wide line detector (*WD*) [19]. These are alternative methods used for image enhancement and they are used separately during evaluation of the system. The proposed *MDT* method is another, alternative method for this sub-block. Next, as binarization, we use the Otsu thresholding [12]. Finally, the generated template of features is matched (e) using Miura match technique [5] against other images in the database (b). The result of this matching (f) is also the final decision of the recognition system.

The image transformation part is very important in the whole pipeline since it is responsible for highlighting the structures within the finger before performing Otsu binarization. The better the structures are revealed in this phase, the more informative features are extracted prior to matching. Here is also the place where we propose our new algorithm, competitive to the other five methods utilized in the pipeline.

## 3. Results of Numerical Experiments

All experiments were carried out on *GustoDB* and with the biometric system described above. The image transformation was performed using five known methods and the proposed *MDT* technique, alternatively. The *GustoDB* was limited to images of the bottom side of fingers, as earlier research [13] has proved that this side allows for reaching a better identification score. The collection of images of a given finger was divided randomly 500 times into the test and database sets. By ’database set’ we understand the set of images used as a reference during the matching phase. In the *GustoDB* we have three images of each finger (images acquired after consecutive removing and putting the finger into the device). Therefore, two images of a given finger were left in the database set while the remaining one was put into the test set. The identification system had to answer the question if the image from the test set belongs to a given person having only two his images in database. The possible answers can be classified into four categories: true positive (*TP*), true negative (*TN*), false positive (*FP*) and false negative (*FN*). To assess the effectiveness of recognition we defined the accuracy (*ACC*), as in our previous work [13]:

(3) $$ACC=\frac{TP+TN}{TP+TN+FP+FN}$$

In such a quality measure all situations encountered by the system are covered: (1) system refused entry to wrong person (*TN*), (2) system refused entry to right person (*FN*), (3) system accepted wrong person (*FP*) and (4) system accepted right person (*TP*). Obviously, the ideal system showing 100% accuracy should have zero false counts (both positive and negative). The *ACC* was calculated for each o 500 trials for all 5 evaluated techniques. Eventually, the mean *ACC* was determined as a final indicator of the identification efficiency.

We also evaluated the false positive ratio (*FPR*) using trails in which a tested person was excluded from the training database. The *FPR*, defined as in Equation (4), indicates the chance that someone is recognised by the system even though he/she is not included in the database. This measure shows the reliability of the system utilized for authentication rather then for identification propose. The results of *ACC* and *FPR* indicators are given in Table 2 and Table 3, respectively.

(4) $$FPR=\frac{FP}{TP+FP}$$

**Figure 5:**
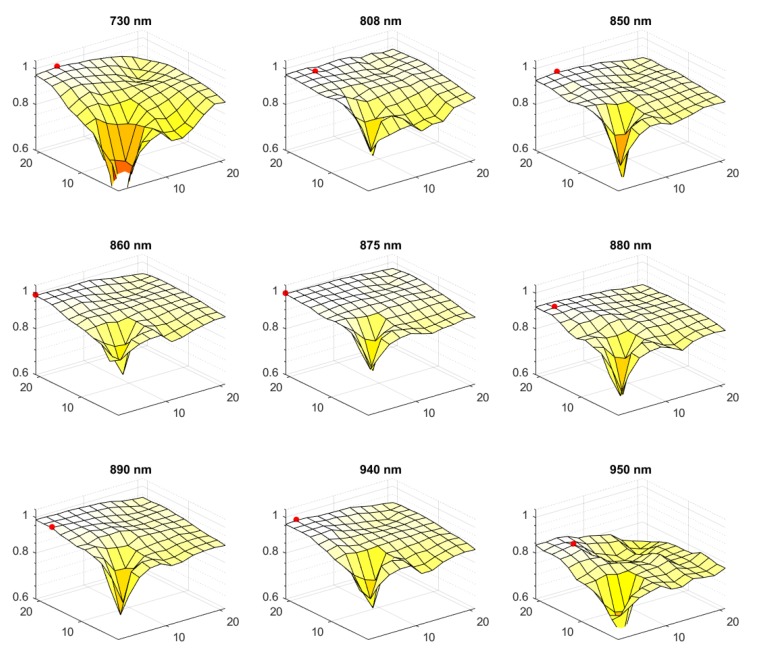
Impact of the window size on verification accuracy. Axis labels for the surface plots are: *x*,*y*—dimensions of the operational window in pixels, *z*—mean *ACC*. The highest values of *ACC* were marked with a red dot.

In the whole evaluation, we modified the parameters of the transformation methods to obtain the highest possible *ACC* values. The *FPR* was not optimized—it was calculated for the best parameters received from *ACC* optimization. In the proposed *MDT* method, this was the size of the running operational window. The best window dimensions indicated by the results of the experiment are listed in Table 2 together with the best *ACCs*. Additionally, the dependencies of the received *ACC* in the function of *x* and *y* dimensions of the window are exposed in Figure 5.

## 4. Disscussion

The experiment proves that the new method introduces a much higher success rate of classification. It can reach 99% in some cases, however, these results should be treated with caution, since it may be biased by a limited number of images/persons used in the experiment. Importantly, the average results higher than 90% for almost all wavelengths are very promising. The analysis of *FPR* confirms a clear advantage of the method as well. In most cases, this measure reaches a magnitude lower false positive rate than other techniques. The second best method in our tests was the wide line detector (*WD*) [19] which managed to outperform the proposed technique for a 950 nm image dataset and was close to its results in other cases. Other methods were not so flexible and universal in terms of utilized wavelength—their efficiency dropped even to 60% and below, in some image sets.

It can be seen that the *MDT* optimization surfaces presented in Figure 5 show clear plateau when the window is large enough to cover the size of a typical structure present in the image. The method’s efficiency decreases when too small window is used (smaller than 4×4, which is approximately 1×1 mm). This indicates that there is very little or even no information hidden in such small structures. It is also clear, that the window should be rectangular with its larger side perpendicular to the finger axis (which is *x* axis in the assumed coordinate system). Such a rectangular window promotes enhancement of features arranged along the finger (mostly the veins). The larger side of the window should have at least 15 pixels (4 mm). However, values larger than 21 pixels (5.5 mm) do not bring any significant improvements. The shorter side should be small, sometimes even the smallest possible 1-pixel. The optimal height should be between 1–5 pixels, which is approximately 0.5–1.5 mm. Summarizing, the desired size of the operational window should be about 5×1 mm on average, for our 3.8 pix/mm sampling rate.

One should also notice the change of plots shape in dependency on the wavelength utilized. While for the shortest and the longest ones (730 nm and 950 nm) the characteristic plateau is not observed and the results are on average poorer, for the light around 860 nm it can be clearly seen having very high mean *ACC* value over wide range of window’s dimensions. This indicates that for this range of NIR light the largest number of characteristic features in the images can be observed and the system’s efficiency is much less dependent on the dimensions of the utilized operational window. Additionally, for the longer wavelengths (950 nm), we could observe that much less external properties of finger skin were extracted after *MDT* enhancement. This was expected as more light is travelling unabsorbed through the finger, lowering the contrast of skin features. This was also accompanied by the lower quantum efficiency of CCD at 950 nm, resulting in poorer signal to noise ratio of the images.

## 5. Conclusions

In the paper, we proposed a new technique for processing of finger vein system images acquired in NIR. Our Modified Distance Transformation (*MDT*), employed before for image quality estimation, appeared to allow for selective enhancing of internal and external structures in the human finger which is crucial in the biometric system prior to the classification phase. The method should be correctly placed within the pipeline used for identification—after preprocessing and before the image binarization stage. By modifying only a single pair of parameters—the size of the operational window—one can easily fit the method to the lens/sensor combination used in a given biometric system.

The comparison with other, widely used processing algorithms, on an extended set of images collected in the *GustoDB* database, showed that our method results in significantly better efficiency of person identification. While our method reached over 90% accuracy, the compared methods showed on average 10% worse results. The tests were performed on a range of NIR images in between 730–950 nm and the superiority of the method was proved over whole this wavelength range. Based on the performed analysis, we also provide the suggested size of the operational window which should be utilized in the *MDT* to optimize its performance. 

## Figures and Tables

**Table 1:** Hardware characteristics of the equipment used for the creation of *GustoDB* biometric database.

Sensor technology	CCD
Image resolution	640 × 480
Image pixel scale	3.8 pix/mm
Illumination type	LED diodes
Wavelengths	730–950 nm

**Table 2:** The mean accuracy *ACC* of Miura match [5] obtained for different segmentation methods: *MDT*, *PGPC*, *MC*, *NAT*, *RDLT*, *WD*. For the new method *MDT*, the best size of operational window was given for each wavelength.

Wavelength	MDT (window)	PGPC	MC	NAT	RDLT	WD
950 nm	85.25 (17×5)	78.70	76.97	67.16	66.66	**87.61**
940 nm	**96.27** (21×3)	77.37	87.76	82.47	83.95	92.23
890 nm	**99.37** (17×1)	86.97	86.10	83.40	84.66	94.92
880 nm	**91.98** (19×3)	79.13	83.89	80.79	81.31	89.92
875 nm	**99.56** (21×1)	87.89	90.48	87.29	90.30	96.43
860 nm	**98.52** (21×1)	85.99	89.52	86.77	89.44	94.78
850 nm	**94.08** (21×5)	79.29	79.60	78.50	78.83	89.92
808 nm	**96.72** (19×5)	79.76	85.67	82.59	83.85	94.05
730 nm	**97.19** (21×5)	62.77	76.09	59.62	62.06	81.82

**Table 3:** The false positive ratio FPR [%] calculated for all compared methods for the best parameters dictated by the *ACC* optimization.

Wavelength	MDT	PGPC	MC	NAT	RDLT	WD
950 nm	0.1966	0.2849	0.3039	0.4386	0.4439	**0.1670**
940 nm	**0.0481**	0.2985	0.1617	0.2318	0.2104	0.1039
890 nm	**0.0175**	0.1716	0.1873	0.2209	0.2000	0.0666
880 nm	**0.1067**	0.2704	0.2150	0.2532	0.2465	0.1326
875 nm	**0.0057**	0.1579	0.1249	0.1649	0.1285	0.0464
860 nm	**0.0199**	0.1818	0.1379	0.1776	0.1381	0.0689
850 nm	**0.0772**	0.2746	0.2685	0.2839	0.2799	0.1324
808 nm	**0.0436**	0.2678	0.1810	0.2364	0.2155	0.0798
730 nm	**0.0379**	0.4913	0.3162	0.5373	0.5056	0.2426
